# Obesity, Dietary Patterns, and Hormonal Balance Modulation: Gender-Specific Impacts

**DOI:** 10.3390/nu16111629

**Published:** 2024-05-26

**Authors:** Elisa Mazza, Ersilia Troiano, Yvelise Ferro, Fabrizia Lisso, Martina Tosi, Ettore Turco, Roberta Pujia, Tiziana Montalcini

**Affiliations:** 1 Department of Clinical and Experimental Medicine, University Magna Græcia, 88100 Catanzaro, Italy; elisamazza@unicz.it (E.M.); tmontalcini@unicz.it (T.M.); 2 Technical Scientific Association of Food, Nutrition and Dietetics (ASAND), 95128 Catania, Italy; ersilia.troiano@gmail.com (E.T.); fabrizia.lisso92@gmail.com (F.L.); 3 Social Educational Directorate of Rome III Montesacro Municipality, 00139 Rome, Italy; 4 Department of Medical and Surgical Science, University Magna Græcia, 88100 Catanzaro, Italy; yferro@unicz.it (Y.F.); roberta.puj@gmail.com (R.P.); 5 “Sant’Anna” Hospital, San Fermo della Battaglia, 22042 Como, Italy; 6 Department of Health Sciences, University of Milan, 20146 Milan, Italy; 7 Department of Public Health, University of Naples “Federico II”, 80131 Naples, Italy; ettoreturco@gmail.com; 8 Research Center for the Prevention and Treatment of Metabolic Diseases, University Magna Græcia, 88100 Catanzaro, Italy

**Keywords:** obesity, gender-specific factors, gender-specific impacts, hormonal balance modulation, endocrine disruptions, dietary patterns, metabolic health

## Abstract

Understanding the intricate relationship between nutrition, hormonal balance, and gender-specific factors is crucial for developing targeted interventions to mitigate obesity-related endocrine disruptions and improve metabolic health. This narrative review examines the impact of various dietary patterns on hormonal regulation in both men and women, focusing on their effects on hormonal balance and metabolic health in the context of obesity. Calorie restriction, the Western diet, high-fat diets, low-CHO diets, plant-based diets, and the Mediterranean diet are analyzed in relation to their influence on obesity-related endocrine disruptions and metabolic health. Future research directions include investigating the specific mechanisms underlying dietary influences on hormonal regulation, addressing the gender-specific metabolic differences and body fat distribution, and exploring the dietary needs of individuals undergoing gender transition. Personalized dietary interventions tailored to individual metabolic and hormonal profiles are essential for optimizing health outcomes across the gender spectrum. By integrating gender-specific considerations into dietary recommendations, healthcare professionals can better support individuals in achieving optimal metabolic health and hormonal balance.

## 1. Introduction

The endocrine system comprises a complex network of cells, tissues, and organs responsible for regulating and coordinating physiological processes through the secretion of hormones [1]. These hormones act on specific target cells within the organs to modulate their function. The endocrine system regulates the appetite and the absorption, storage, and utilization of nutrients, as well as other functions, including reproduction [1,2,3]. The glands constituting the endocrine system include the pituitary, thyroid, parathyroid, adrenal, thymus, and pineal, as well as the pancreas, ovaries, and testes [1,2]. Although more than 200 hormones can be found within the human body, including estrogen, testosterone, cortisol, insulin, leptin, and thyroid hormones, they are predominantly recognized for their close association with the metabolism, fertility, mood regulation, and other essential physiological functions [1,2,3].

Specific nutrients and dietary patterns can either positively or negatively influence hormonal balance [3]. Dietary intake patterns, including excessive caloric intake or fasting and foods with high glycemic loads (GLs), are known to affect circulating hormone levels [2]. For instance, the regulation of thyroid hormones is modulated by the physical metabolic condition, and a sufficient intake of essential nutrients such as selenium, iron, and iodine is essential to preserve the optimal thyroid hormone activity [4]. Furthermore, a recent meta-analysis highlights that obesity is significantly associated with an increased risk of hypothyroidism and Hashimoto’s thyroiditis and increased levels of positive thyroid peroxidase antibody (TPOAb) [5]. Additionally, cortisol, a steroid hormone, regulates various physiological processes and is influenced by dietary extremes and specific nutrients, such as the type of CHOs consumed [6].

Dietary patterns and, thus, nutritional composition can also impact the sensitivity of tissues to specific hormones. A study comparing two dietary patterns (a Mediterranean diet vs. a low-carbohydrate diet) found that both patterns improved insulin resistance (IR) and β-cell glucose sensitivity, independently from glycemia and weight loss [7]. Leptin, released from adipose tissue, can be modulated by specific dietary habits and nutritional intake [8]. Anti-inflammatory diets, such as the Mediterranean diet, enhance leptin sensitivity and decrease leptin levels, while diets high in saturated fatty acids (SFAs) can induce leptin resistance, potentially leading to obesity [9].

Obesity is a multifactorial condition influenced by individual and environmental factors, encompassing dietary quality, detrimental eating behaviors, and inadequate physical activity levels [10]. Obesity is known to impact the function of the endocrine system, with dysfunctional adipose tissue being a key contributor to this effect [11]. Excessive lipid storage in the adipocytes results in various metabolic changes, including mitochondrial dysfunction and subsequent endoplasmic reticulum stress [12]. These metabolic shifts can alter the secretion profile of adipose tissue-derived substances, known as adipokines, leading to a low-grade inflammation status. The adipokines exert endocrine effects and influence tissues and organs systemically, including the endocrine glands [13,14].

The World Health Organization (WHO) indicates an increasing prevalence of obesity in developing nations, with a higher incidence observed among women worldwide compared to men (40% vs. 35%) [15]. According to the 2021–2022 surveillance system PASSI (Progresses in ASSessing adult population health in Italy), approximately 43% of Italian adults are affected by overweight or obesity, exhibiting a north–south gradient in obesity prevalence. Notably, in Italy, overweight and obesity are more prevalent among men than women, with total rates of 52% and 34%, respectively [16]. The disparity in obesity prevalence between genders across countries implies that, beyond biological and behavioral determinants, socio-economic factors may also contribute to this phenomenon. The difference in the prevalence and growth rate of obesity between males and females has prompted numerous researchers to explore the gender-specific pathogenic mechanisms and phenotypic characteristics of obesity, aiming to discern gender-tailored weight loss strategies [17], especially considering the increasing prevalence of infertility in couples, attributed to both genders. Overweight women face reduced ovulation and lower rates of spontaneous conception [18]. They also show a high risk of miscarriage and adverse pregnancy outcomes [18]. Furthermore, obesity can have a negative impact on male fertility through endocrine, thermal [18], genetic [19] and sexual [18] pathways.

This narrative review aims to investigate the relationship between body composition and nutritional impact on hormonal regulation and obesity, focusing on gender differences. It explores the impact of dietary patterns and adipose tissue dysfunction on endocrine function, highlighting gender disparities and the need for further research on gender transition for understanding the complex interplay between hormones, metabolism, and dietary patterns.

## 2. Body Composition and Gender Specificity in Body Fat Distribution

Extensive evidence indicates that gender-specific differences in biochemistry and physiology are influenced not only by exposure to cyclical hormonal patterns but also by inherent biological factors [20]. In terms of body composition, while the average body fat percentage is similar between males (12%) and females (15%), the latter have four times the total adipose tissue volume compared to males, accounting for 12% versus 4% of the total body weight [17]. This essential adipose tissue (Essential Fat, EF) is vital for pregnancy and hormonal functions, ensuring the optimal physiological functioning. A marked reduction in EF, resulting from extreme dietary regimens or intense physical activity, can significantly risk overall health status [18,21]. In females, EF also encompasses “sex-specific fat”, representing about 5–9% of the total body fat and localizing in the breast, genital regions, lower body subcutaneous tissue, and intramuscular deposits [18]. In addition to the total body fat mass, a significant difference is evident in the distribution of body fat deposits between males (abdominal or “apple-shaped”) and females (gluteofemoral or “pear-shaped”) [22]. In males, the abdominal fat tissue tends to accumulate more in the visceral area compared to females [23,24], and, with an equivalent fat mass, males typically have about twice the visceral fat accumulation than females [25]. These differences reflect variations in the endocrine status, including estrogens, androgens, the growth hormone (GH), and insulin-like growth factor 1 (IGF-1), which emerge from the prepubertal phase onwards [18]. The extent of sexual differences intensifies with maturation, particularly from late puberty to early adulthood, as males develop a more android body shape and females a more gynoid one [26]. This clearly underscores the influence of sexual hormones. Available data suggest a clear gender difference in age-related changes in the body fat distribution, especially in abdominal adipose tissue, with a marked increase in visceral fat accumulation occurring after menopause [18,26,27]. Indeed, after the menopause, women exhibit a propensity to accumulate more fat in the visceral depot, reflecting the pattern typically observed in men [27]. Men tend to exhibit greater visceral adiposity, leading to adverse metabolic outcomes like increased postprandial insulin, free fatty acids, and triglycerides [28]. In contrast, the peripheral adipose tissue distribution commonly seen in women is less linked to obesity-related complications compared to the central adipose distribution [28]. The accepted consensus regarding the increased risk of complications from adiposity indicates that this risk increases when adipose mass exceeds a certain threshold, estimated at approximately 25% in males and 30% in females [26,28]. This is predominantly influenced by an obesogenic environment, characterized by energy-dense foods, high sugar intake, societal structures limiting physical activity, and easily accessible, low-cost sedentary behaviors such as watching television, playing video games, and surfing social media [29,30,31]. It has recently been demonstrated that the composition of gut microbiota influences fat accumulation by affecting the expression of the key transcription factors and proteins involved in fat synthesis and the metabolism, such as ChREBP, SREBP-1, and FIAF/ANGPTL4, thereby impacting fat storage and potentially promoting obesity [32]. The complications associated with obesity significantly increase with elevated visceral adiposity [33]. These commonly include cardiovascular diseases (CVDs), type 2 diabetes (T2D), various cancers, accelerated aging, neurodegeneration, and detrimental effects on human reproduction [33,34]. These complications are thought to be mediated by complex and not fully understood mechanisms, encompassing IR and promoting hyperinsulinemia, elevated leptin levels, chronic low-grade systemic inflammation, and oxidative stress (OS) [33,34]. Furthermore, scientific evidence demonstrates that the accumulation of body fat is associated with infertility in both men and women due to various underlying mechanisms [35]. Currently, obesity is a recognized contributing factor to infertility in approximately one out of seven couples [35]. Gender differences in body composition and fat distribution, shaped by both hormonal influences and inherent biological factors, highlight the need for gender-specific approaches in understanding and managing adiposity-related complications and associated health risks.

## 3. Obesity and Hormonal Imbalances in Males

Obesity is associated with adipocyte hypertrophy and hyperplasia, which lead to changes in endocrine regulation in men, primarily through the secretion of adipokines [34]. These physiological changes detrimentally impact the male reproductive endocrine system, primarily via the Hypothalamic–Pituitary–Gonadal (HPG) axis [36].

Hypogonadism in men with obesity represents a multifactorial pathological condition characterized by altered gonadal function and androgen deficiency. The prevalence rates of male hypogonadism have shown an upward trend over the past decade, often being underestimated and underdiagnosed [37]. In obesity-related hypogonadism, excess body fat and elevated levels of leptin play crucial roles. This pathological state not only impairs fertility and sexual function but also influences bone mineralization, fat metabolism, cognitive function, muscle mass, and body composition [37]. The pathogenesis of male obesity-related secondary hypogonadism (MOSH) involves three major risk factors: hypoestrogenism, metabolic endotoxemia, and hyperleptinemia [37,38]. Adipose tissue expansion, driven by weight gain, leads to increased aromatase activity, converting testosterone to estradiol and suppressing the secretion of LH [39]. Metabolic endotoxemia, facilitated by dietary habits, compromises testicular function through the inflammatory responses triggered by bacterial endotoxin. Moreover, elevated leptin levels in obesity inhibit androgen production, exacerbating hypogonadism in affected individuals [40].

A negative association exists between free thyroxine (T4) levels and Body Mass Index (BMI) [41]. Furthermore, leptin is now acknowledged as a pivotal endocrine mediator in various physiological functions, including reproduction. Since the identification of thyroid hormone and leptin receptors within testicular tissues, it has been recognized that these hormones are implicated in male reproductive physiology [40,41]. A relationship is discernible between thyroid hormones, leptin, and reproductive function, with potential adverse effects on spermatogenesis leading to male infertility [41,42]. Moreover, men with obesity experience physical conditions like erectile dysfunction and elevated scrotal temperature, both of which are additional factors contributing to male infertility [42].

*Hyperinsulinemia, leptin, and insulin resistance (IR).* Individuals with obesity frequently exhibit elevated insulin levels as a result of IR, which may induce genetic and epigenetic alterations, potentially influencing male reproductive endocrine regulation and fertility. [43]. In T2D, secondary hypogonadism may result from both peripheral and central IR, aggravated by pro-inflammatory cytokines like Tumor Necrosis Factor Alpha (TNF-α) and Interleukin 6 (IL-6) that influence the HPG axis. The elevated insulin levels associated with obesity correspond with decreased levels of the Sex Hormone Binding Globulin (SHBG) in men [44]. Testosterone is a steroid hormone primarily produced in the testes, though smaller amounts are also synthesized in the adrenal glands [45]. It plays a critical role in various physiological processes, including the development and maintenance of male reproductive tissues such as the testes and prostate [45]. Additionally, testosterone is essential for the development of secondary sexual characteristics during puberty, including the deepening of the voice, the growth of facial and body hair, and muscle development. Beyond its roles in reproduction and sexual functions, testosterone also impacts bone density, fat distribution, muscle mass, and red blood cell production [45,46]. Low testosterone levels are associated with both IR and obesity, suggesting the independent impact of IR on testosterone synthesis, even when accounting for SHBG levels [44]. The increased leptin secretion in men with obesity can inhibit testosterone production by Leydig cells [35]. Excessive adipose tissue promotes testosterone conversion to estradiol through increased aromatase activity [44]. Parameters such as BMI, total body fat percentage, and intra-abdominal fat correlate inversely with testosterone and positively with estrogen levels in males [47]. Reduced sperm quality is common in men with obesity [48], and between 20% and 64% of such men have decreased total testosterone levels. Erectile dysfunction and low sperm count are prevalent in this group [48]. Men with T2D and obesity often show reduced total and free testosterone levels and lower SHBG levels [49]. Calderón et al. [50] found that, with moderate to severe obesity, decreased ejaculate volume and testosterone levels were inversely related to IR, BMI, and excessive body weight. Obesity can enhance testosterone conversion to estradiol, suppressing the reproductive axis and leading to secondary hypogonadism [50]. Another study demonstrated a correlation between obesity and elevated insulin levels, which have been associated with adverse effects on key fertility indicators such as sperm quality and reproductive hormone levels [51].

*Cortisol*. Cortisol, the primary glucocorticoid in humans, is intricately involved in the stress response and various physiological processes. Its implications for the abdominal fat accumulation and increased appetite are noteworthy [52]. The contemporary surge in obesity aligns with the factors promoting cortisol production, such as chronic stress and sleep deprivation, suggesting a cyclic relationship where heightened glucocorticoid activity, obesity, and stress reciprocally reinforce each other. Recent studies highlight the significant associations between obesity and long-term cortisol levels, measured in scalp hair, across diverse age groups [53,54]. The chronic exposure to elevated glucocorticoid levels can lead to abdominal obesity, metabolic syndrome, and cardiovascular diseases (CVDs) [52]. In males, chronic stress and heightened cortisol levels correlate with visceral fat accumulation, insulin resistance, and an elevated risk of metabolic syndrome. Elevated cortisol levels in men with obesity, often influenced by stress, signify hypothalamic–pituitary–adrenal (HPA) axis dysregulation [55]. This dysregulation results in heightened cortisol secretion, particularly in the morning and postprandially, contributing to abdominal obesity and metabolic irregularities. Additionally, high cortisol levels may impact other hormone systems, including reductions in insulin-like growth factor I (IGF-I) and testosterone levels [55].

*Thyroid hormones*. Obesity is associated with alterations in thyroid function, including increased thyrotropin (TSH) and active triiodothyronine (T3) levels, without significant changes in T4 [56]. These changes suggest an influence on the Hypothalamus-Pituitary-Thyroid (HPT) axis and potential neuroendocrine dysfunction [57]. Furthermore, obesity is associated with dysregulated leptin production, which may have additional effects on TSH regulation, such as through mechanisms involving leptin resistance [58]. These alterations point to disrupted energy balance and hormone resistance in obesity. In men with obesity, studies have shown an increase in TSH levels associated with a slight decrease in T3, while T4 levels generally remain unchanged [59]. This suggests reduced thyroid activity and a potential state of thyroid hormone resistance. The diminished tissue responsiveness to thyroid hormones may result from reduced signaling of both TSH and thyroid hormones within adipocytes. Consequently, the body may increase TSH and fT3 secretion to compensate for this alteration [60]. Additionally, a positive correlation between BMI and TSH levels has been observed in both men and women, but it is more pronounced in men [59].

*Dyslipidemia*. In obesity and its effects on hormonal imbalance, dyslipidemia plays a significant role. An imbalance in dietary lipids can involve fatty acids, cholesterol, or both. The main effect of dyslipidemia is the development of systemic OS, which is known to impact the quality of male germ cells and is associated with infertility. Excessive intake of a diet high in cholesterol or fats (HFD) induces hypercholesterolemia and/or hyperlipidemia, detrimental to fertility and the proper functioning of male reproductive organs [18,46]. Hyperlipidemia modifies the structures and functions of testes and epididymis, impacting on the energy metabolism and the process of spermatogenesis. This condition initiates OS, compromising the male reproductive system. Consequently, it triggers apoptosis, reduces sperm concentration, motility, and viability, increases morphological abnormalities, and induces sperm DNA fragmentation. Moreover, hyperlipidemia hampers acrosome response and capacitation, induces hormonal imbalances in males and leads to erectile dysfunction [18,46].

*Oxidative stress and inflammation*. Obesity is a clinical manifestation of systemic OS, characterized by elevated levels of proinflammatory cytokines such as TNF-α and IL-6, which contribute to chronic low-grade inflammation [51]. OS results from an imbalance between the reactive oxygen species (ROS) production and the body’s antioxidant defense mechanisms. This imbalance not only affects fertility but also influences various metabolic and hormonal pathways [42]. OS exerts its detrimental effects on male fertility through various pathways. Elevated ROS levels in the sperm impair membrane integrity, reduce motility, and compromise DNA integrity, potentially impacting embryo development and leading to premature cell death and decreased quality [42,61]. Additionally, OS contributes to IR and the dysregulation of other hormones like leptin [58]. Elevated ROS levels can lead to increased inflammation and IR, exacerbating the metabolic dysfunction commonly seen in obesity [58].

*Microbiota.* Alterations in the composition of the gut microbiota (GM) play a pivotal role in obesity and inflammation. The GM plays a significant role in obesity-related hormonal imbalances through the absorption of lipopolysaccharides (LPS) from Gram-negative bacteria [62]. The increased LPS levels trigger chronic low-grade inflammation, promoting obesity and IR [62]. This inflammatory response is mediated by substances like TNF-α, IL-6, and the leptin released from adipose tissues. Changes in the GM are linked to obesity development, with distinct differences observed between men and women [63]. In men with obesity, the Bacteroides genus is reduced, while *Veillonella* and *Methanobrevibacter* are more prevalent compared to women. Conversely, *Bilophila* is less abundant in men. After accounting for age and gender, 66 bacterial taxa correlated with BMI and plasma lipid levels. Cox et al. [64] revealed that the changes in gut microbiota influence intestinal permeability, triggering immune signaling and contributing to the chronic low-grade inflammation that is linked to obesity-related diseases. Emerging evidence highlights the importance of intestinal microbial eubiosis for reproductive health, with GM alterations potentially leading to reproductive diseases [65]. Furthermore, the modulation of GM has been shown to influence the development of the blood–testis barrier and intra-testicular testosterone levels, potentially influencing the development and maturation of the Sertoli cells [66].

## 4. Obesity and Hormonal Imbalances in Females

Increases in body weight and adipose tissue are associated with abnormalities in sexual steroid levels in both premenopausal and postmenopausal women [67]. Central obesity in women has been shown to result in higher circulating levels of androgens, even in the absence of a clinical diagnosis of Polycystic Ovary Syndrome (PCOS) [67]. These women exhibit higher total and free testosterone levels compared to normal weight women, but lower levels of androstenedione and SHBG [67]. Imbalances in androgens, specifically hyperandrogenemia, in women are linked to metabolic diseases and obesity [68]. In fact, excessive androgen levels in women with PCOS are associated with IR and obesity [68]. The fertility of women with PCOS may be compromised through several mechanisms, including infrequent ovulation or anovulation, a heightened risk of miscarriage, a diminished oocyte quality, insulin resistance resulting in hyperinsulinemia and an increased risk of miscarriage, and prolonged intimal hyperplasia, which can impair implantation [69]. PCOS is a prevalent condition marked by hyperandrogenism and frequently linked to obesity, impacting around 6–10% of women of reproductive age [70]. About two-thirds of women with PCOS are obese, and 50–70% of them show IR [71]. PCOS emerges as the primary causative factor for hyperandrogenism and oligo-anovulation during the reproductive years, often entwined with infertility, as well as clinical and metabolic comorbidities [72]. The prevalence of infertility among individuals diagnosed with PCOS typically falls within the range of 70 to 80% [73]. Research findings indicate that overweight and obese individuals diagnosed with PCOS could experience notable enhancements in fertility, the metabolic parameters, and psychological well-being by achieving a weight loss ranging between 5% and 10% [74,75].

The molecular mechanisms underlying the sex-specific effects of testosterone remain largely unexplored. While testosterone itself is not detrimental to the female metabolism, it may interfere with essential neuroendocrine functions. Elevated testosterone levels can modify the ovarian morphology and function, diminishing the protective effects of female hormones against the metabolic stress induced by overnutrition [76]. Furthermore, the tissue distribution and activation states of the peripheral and central androgen receptors may differ between males and females. Long-term testosterone administration in female-to-male transgender individuals results in weight gain and visceral obesity [77], suggesting that androgens may selectively stimulate visceral white adipose tissue hypertrophy and/or hyperplasia, thereby increasing the risk of IR and metabolic syndrome in hyperandrogenic women.

Female obesity manifests as irregular menstrual cycles, increased miscarriage rates, anovulation, and adverse neonatal and maternal outcomes [78]. An elevated BMI may also induce ovulatory dysfunctions in women and is correlated with conditions like hirsutism and hormonal imbalances, affecting menstrual patterns [79]. Obesity modifies the natural ovulation process [80] and disrupts the hormonal balance, potentially compromising the oocyte quality and development [81]. Furthermore, obesity is associated with prolonged gestational periods and gestational diabetes [82]. Moreover, elevated adipose tissue levels in women exacerbate PCOS due to increased detrimental adipokine levels and decreased beneficial adipokine concentrations [83].

Recent findings have unveiled the concept of “female obesity-related secondary hypogonadism”, analogous to its male counterpart, underscoring its importance among women with obesity [84]. Obesity correlates with reduced LH levels, due to heightened LH clearance and reduced pituitary responsiveness to gonadotropin-releasing hormone (GnRH). In women with obesity, particularly those with PCOS, the LH pulse amplitude decreases, worsened by elevated androgen levels [85]. Moreover, the elevated leptin levels induce hypothalamic resistance to leptin, further dampening the GnRH pulsatility and LH secretion. Additionally, anti-Müllerian hormone levels in women with obesity remain stable or reduced, potentially decreasing following weight loss [84].

In summary, obesity significantly influences hormonal imbalances in females, affecting sexual steroid levels and reproductive health outcomes. Understanding these interconnections is crucial for targeted interventions and management strategies to mitigate the adverse effects of obesity on female reproductive health.

*Thyroid hormones.* Thyroid hormones play a pivotal role in regulating metabolism and energy balance, and their dysfunction can have significant clinical implications. In women with obesity, thyroid function can be influenced by various factors, including hormonal fluctuations associated with the menstrual cycle and menopausal status [58,59]. Studies have demonstrated that women with obesity often exhibit increased variability in TSH levels throughout the menstrual cycle, with elevated peaks observed during the luteal phase [57,59]. This fluctuation in TSH levels may be closely correlated with changes in leptin concentrations [86]. Leptin plays a crucial role in regulating the hypothalamus-pituitary-thyroid (HPT) axis. Changed leptin levels can thereby induce modifications in the HPT axis function, potentially leading to thyroid dysfunction [57]. Thyroid dysfunction in women with obesity can exacerbate metabolic disturbances, contributing to IR, dyslipidemia, and cardiovascular complications [87]. Additionally, altered thyroid function may further complicate weight management efforts, as thyroid hormones influence energy expenditure and fat metabolism [88]. Therefore, understanding the intricate relationship between obesity, leptin, and thyroid function is essential for comprehensive management strategies and targeted interventions to improve metabolic health in women with obesity.

*Hyperinsulinemia, leptin, and insulin resistance (IR).* Women of reproductive age demonstrate greater insulin sensitivity in the skeletal and hepatic muscles, increased stimulated insulin secretion, and, consequently, lower fasting glucose and HbA1c levels compared to men [89]. However, during menopause, blood pressure and the levels of LDL cholesterol and HbA1c rise, alongside unfavorable changes in the body fat distribution [4], contributing to impaired glucose tolerance (IGT) [89]. Obesity in women is associated with hyperinsulinemia and IR, which significantly contribute to infertility and impacts ovarian function. Insulin stimulates the theca cells in the ovaries to produce androgens, thereby promoting the synthesis of estradiol and influencing steroidogenesis through the activation of its receptor [83]. In the ovary, leptin and its receptor (LEP-R) in the granulosa cells regulate ovulatory functions and gonadal activity [90]. Obesity-induced high leptin levels can negatively impact androstenedione synthesis in the ovarian cells and disrupt oocyte development and interaction with the surrounding cells [91]. Elevated leptin levels can also impair ovulation through mechanisms independent of plasma female sex hormone levels [90]. In the context of PCOS, IR leads to hyperinsulinemia. While the liver, ovaries, and adrenal glands remain insulin-sensitive, the skeletal muscle and adipose tissue develop IR. This results in a reduced glucose uptake and increased lipolysis. The heightened insulin levels stimulate the ovaries and adrenal glands to produce more androgens, contributing to follicular arrest and anovulation. Additionally, hyperinsulinemia inhibits the liver’s production of SHBG, leading to increased testosterone levels in the blood [92]. Hyperinsulinemia exerts various effects in individuals with PCOS, negatively impacting the pituitary gland and enhancing the LH pulse amplitude, increasing sensitivity to the gonadotropin-releasing hormone [93]. It stimulates the activity of the P450c17α enzyme in the adrenal cells, further increasing the adrenal androgen production. Elevated insulin and LH levels activate the P450c17α enzyme in the ovarian theca cells, leading to increased ovarian androgen production and subsequent hyperandrogenism [94]. Additionally, hyperinsulinemia reduces SHBG levels, thereby increasing the free androgens. It may contribute to hyperandrogenism through obesity-induced metabolic alterations, such as the increased release of free fatty acids from the adipose tissue [83]. Obesity exacerbates hyperandrogenism in women with PCOS, with a higher prevalence of hirsutism and menstrual irregularities in women with obesity compared to those of normal weight. Moreover, obesity modifies the lipid profile, increasing cardiovascular risk; however, reversing insulin resistance may mitigate these metabolic complications [93,94]. Furthermore, obesity-related hyperinsulinemia and IR are also linked to menstrual irregularities, hyperandrogenism, and alterations in steroidogenesis in both women with obesity and of a normal weight [93]. In summary, hyperinsulinemia resulting from obesity and IR detrimentally impacts ovarian function and reproductive health in women by disrupting steroidogenesis. This disruption can contribute to the development of conditions such as PCOS and infertility.

*Cortisol.* Women with obesity may be particularly susceptible to the effects of cortisol on metabolic and hormonal health. In female obese individuals, elevated cortisol levels have been associated with visceral fat accumulation, IR, and an increased risk of metabolic syndrome [95]. In premenopausal women with obesity, cortisol secretion and its effects on metabolic and hormonal health are of particular interest. Studies have shown that women with obesity with an elevated waist-to-hip circumference ratio (WHR)—especially those with increased abdominal sagittal diameter indicative of visceral fat accumulation—exhibit higher urinary cortisol output under field conditions. Interestingly, despite elevated cortisol levels, the dexamethasone inhibition of cortisol secretion remains normal in these individuals [96]. Furthermore, responses to stimulatory tests, including exposure to physical or mental stressors, also result in heightened serum cortisol levels. Importantly, these responses are not mirrored in prolactin or growth hormone concentrations [96]. These findings suggest that women with obesity with visceral fat accumulation may have increased cortisol secretion due to heightened sensitivity along the HPA axis. This dysregulation could potentially contribute to their abnormal fat depot distribution and metabolic abnormalities.

*Oxidative stress, microbiota, and inflammation.* In women with obesity, an increased risk of reproductive issues due to systemic OS and inflammation was found. This is characterized by the elevated levels of inflammatory cytokines like TNF-α and IL-6, which are commonly found in obesity-induced chronic inflammation [97]. The imbalance between the production of ROS and antioxidant defenses can compromise follicle development, oocyte maturation, and both embryo and placental growth [98]. This can lead to lipid peroxidation, cellular damage, and apoptosis. Moreover, disruptions in leptin pathways during pregnancy can impede oocyte development and its interaction with adjacent cells, further complicating reproductive outcomes [82]. Additionally, metabolic abnormalities in oocytes associated with obesity, such as elevated intracellular lipid levels, heightened OS, and mitochondrial dysfunction, are connected to both systemic and localized inflammatory responses [98]. These changes can result in menstrual irregularities, infertility due to endometriosis, intrauterine growth restriction, preeclampsia, recurrent miscarriages, and premature births [99]. Alterations in the composition of the gut microbiota has been linked to the development of various metabolic disorders in women, including PCOS [100]. Several studies have reported alterations in the overall composition of gut bacteria in women with PCOS, showing a decrease in both species’ diversity within a sample (alpha diversity) and similarity between samples (beta diversity) [100]. Furthermore, the genital microbiome plays a crucial role in female fertility [101]. Dysbiosis and *Lactobacillus* deficiency can interfere with assisted reproduction treatments and partially explain implantation failures. A balanced presence of *Lactobacillus* species, particularly *Lactobacillus crispatus*, is protective against asymptomatic bacterial vaginosis (BV), which is primarily maintained by *Ureaplasma* and *Gardnerella vaginalis* and is recognized as potentially affecting fertility [101,102]. Moreover, the presence of Gram-negative bacteria, *Chlamydia trachomatis*, and *Gardnerella vaginalis* in the cervical flora, along with a possible deficiency of *Lactobacillus*, has been associated with infertility problems [101]. Davis [103] reported that correlations between obesity, changes in gut microbiota composition, ovarian inflammatory responses, and oocyte gene expression may contribute to infertility and other reproductive disorders in women [103]. Therefore, the OS and inflammation linked to obesity significantly impact female reproductive health by compromising oocyte quality, follicular development, and overall reproductive success. The proper regulation of the inflammatory signaling pathways is essential for maintaining normal hormonal balance, and any dysregulation can contribute to various endocrine disorders [97,98,99].

**Figure 1:**
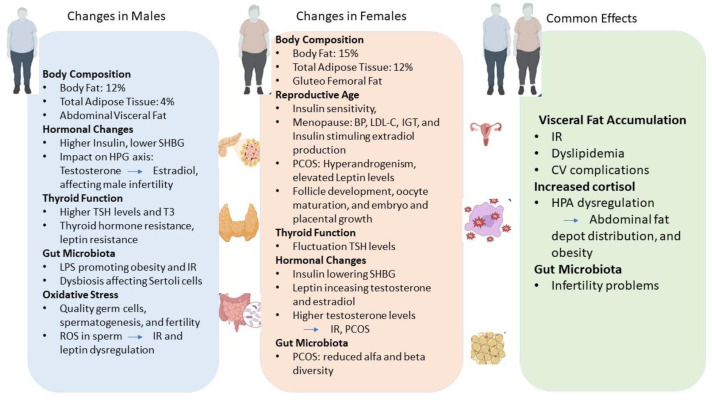
Graphical representation of the main differences in terms of hormonal and endocrine changes in males and females and common effects due to obesity. Abbreviations: IR—insulin resistance; PCOS—Polycystic ovary syndrome; HPG—hypothalamic–pituitary–gonadal; TSH—Thyroid-stimulating hormone; CV—cardiovascular; T3—Triiodothyronine; LPS—Lipopolysaccharide; ROS—reactive oxygen species; SHBG—Sex hormone-binding globulin; BP—blood pressure; LDL-C—low-density lipoprotein—cholesterol; IGT—impaired glucose tolerance; and HPA—hypothalamic–pituitary–adrenal. → leads to.

Table 1 summarizes the body composition and fat distribution differences between men and women, as well as the main endocrine and hormonal imbalances resulting from obesity in both sexes. Figure 1 illustrates the main differences in terms of hormonal and endocrine changes in males and females, as well as the common effects due to obesity.

## 5. Nutritional Influences on Hormonal Balance and Gender-Specific Implications for Obesity-Related Endocrine Disruptions

Nutrition is increasingly acknowledged as a pivotal environmental determinant influencing endocrine function, reproductive health, and obesity [104]. As the precursors of essential molecules governing bodily processes, nutrients play a crucial role in modulating physiological functions and biochemical pathways, including those regulating hormones [45,104]. The significant impact of nutrition on obesity underscores its central role in overall metabolic health in both genders. In contemporary nutritional research, it is also crucial to assess overall dietary patterns rather than focusing solely on individual foods or nutrients [105]. This holistic approach is essential because modern diets are characterized by the consumption of a diverse array of foods throughout the day, each contributing in unique ways to overall health and metabolic outcomes [105]. Different dietary patterns have distinct effects on hormonal regulation, which can be particularly relevant in the context of obesity-related endocrine disruptions. Understanding these influences is crucial for developing targeted dietary interventions for obesity management. Furthermore, emerging research suggests the potential transgenerational effects of diet. For example, studies have indicated that dietary cues can influence sperm signatures and oocyte quality [106], thus impacting reproductive outcomes across generations.

Calorie restriction is gaining increasing attention among researchers, and its effects on health, including hormonal balance, are being more comprehensively evaluated [107]. This interest stems from growing evidence suggesting that a reduction in the energy intake can exert beneficial effects on various physiological processes, influencing not only weight management but also overall metabolic health and hormonal regulation [107]. Gender-specific differences in nutritional requirements and responses to dietary interventions are increasingly recognized. Men and women may have distinct hormonal responses to different dietary patterns and caloric intakes, which can contribute to gender-specific obesity-related endocrine disruptions [108]. Therefore, understanding the intricate interplay between nutrition, hormonal balance, and gender-specific factors is crucial for developing targeted dietary interventions and therapeutic strategies to mitigate obesity-related endocrine disruptions and to improve metabolic health in both men and women. To delve deeper into these gender-specific differences, the following sections will explore the distinct hormonal and metabolic responses to nutrition and dietary patterns observed in men and women.

## 6. Nutritional Impact on Hormonal Regulation

Both males and females with overweight or obesity are at an elevated risk of disrupted hormonal regulation and increased ROS production [109]. Imbalanced dietary regimens can precipitate a state of low-grade systemic inflammation, profoundly affecting endocrine homeostasis [109]. Nutrition is a critical determinant of hormonal balance in both sexes, with dietary patterns exerting significant influences on reproductive health and endocrine function. In females, nutritional factors are pivotal in regulating hormonal balance, impacting parameters such as menstrual cycles, ovulation, and fertility [21,22,26] Similarly, in males, dietary intake plays an integral role in maintaining hormonal equilibrium and sustaining optimal reproductive function [109].

Calorie restriction and weight loss. Calorie restriction (CR) has been widely recognized as a potent approach to achieve weight loss [110]. The extent of this restriction can range from an approximately 25% reduction in caloric intake to a very-low-calorie diet (VLCD) with an absolute intake of 400–800 kcal/day [111]. Numerous in vivo and human studies suggest that CR in overweight and obese patients may improve hormonal balance [112,113]. A meta-analysis shows that in overweight and obese humans undergoing CR, fat loss was found to be sex- and age-specific: younger females (<45 years) showed resistance to fat loss compared to their male counterparts, whereas this difference was negligible in older individuals (>45 years) [12]. These findings highlight the age-dependent sex variations in CR’s metabolic effects and emphasize the roles of adipose tissue, liver function, and estrogen levels in determining CR’s metabolic benefits [112]. A systematic review and meta-analysis demonstrated that while CR significantly increases serum cortisol levels initially, this effect diminishes over time, indicating the transient activation of the HPA axis [114]. Fasting had a pronounced effect on increasing cortisol levels, while VLC and less intense LC diets did not show significant increases, suggesting the differential effects of various forms of calorie restriction on cortisol dynamics in both genders [114].

A recent meta-analysis indicates that CR elevates testosterone concentrations in overweight or with obesity men, while reducing testosterone levels in healthy men with a normal body weight [115]. In men with overweight and obesity, weight loss primarily acts by reducing adipose tissue, inflammation, and improving insulin sensitivity, leading to increased testosterone production. In contrast, weight loss in men with a normal weight may decrease testosterone levels due to increased cortisol concentrations, reduced leptin levels, and the effects of other hormones like ghrelin and adiponectin [115]. Hence, CR modulates testosterone concentrations in men, with the effect being BMI-dependent. These findings are supported by another meta-analysis [116], indicating that the beneficial changes in testosterone levels observed with a ketogenic diet (KD) are more likely due to the induced weight loss rather than the ketogenic metabolic state per se [116]. Essentially, CR and the subsequent weight reduction appear to be the predominant factors influencing the increase in testosterone levels, rather than specific effects of the ketogenic diet. On the other hand, a 10-day fast in men with obesity led to a significant decrease in serum testosterone levels, which normalized during re-feeding [117]. Malnutrition and protein restriction or protein–energy deficiency may impair Leydig cell function and testosterone biosynthesis [118].

CR and dietary correction can significantly improve metabolic syndrome and reduce IR [119]. However, these interventions may not fully reverse fertility-related health issues. Testicular metabolic memory can result in persistently lower sperm quality despite improved metabolic health [120]. This involves lasting epigenetic changes and oxidative stress in the testes, impairing spermatogenesis [121].

CR can negatively impact sperm quality by inducing metabolic and hormonal changes. CR leads to a metabolic shift from glucose-dependent to lipid-dependent pathways, activating AMPK and inhibiting the mTOR pathway [122]. This metabolic alteration reduces protein synthesis and increases autophagy, which, while beneficial for cellular health, can impair spermatogenesis [122]. Additionally, CR influences the hormonal balance by increasing cortisol levels and altering the secretion of gonadotropins, which can disrupt the HPG axis [114]. This hormonal imbalance, coupled with reduced energy availability, can decrease testosterone production and impair the function of the Sertoli and Leydig cells, essential for healthy sperm production. Furthermore, CR-induced epigenetic changes can modify gene expression patterns critical for sperm development, potentially leading to reduced sperm quality [123,124].

The available data indicate that a 5% weight loss in women with obesity results in significant improvements in endocrine parameters, including decreased levels of free testosterone, LH, and insulin, as well as increased ovulation frequency [125]. Studies indicate that weight loss could potentially normalize abnormal sex hormone levels in obese premenopausal women and those with hyperandrogenism [113,126]. Specifically, visceral fat reduction has been found to be significantly associated with decreased androgen levels in premenopausal women with obesity, but not in men with obesity [113]. Turcato et al. [113] demonstrated that weight loss primarily affects the hormones in premenopausal women while no significant hormonal changes were observed in postmenopausal women after weight loss.

**Figure 2:**
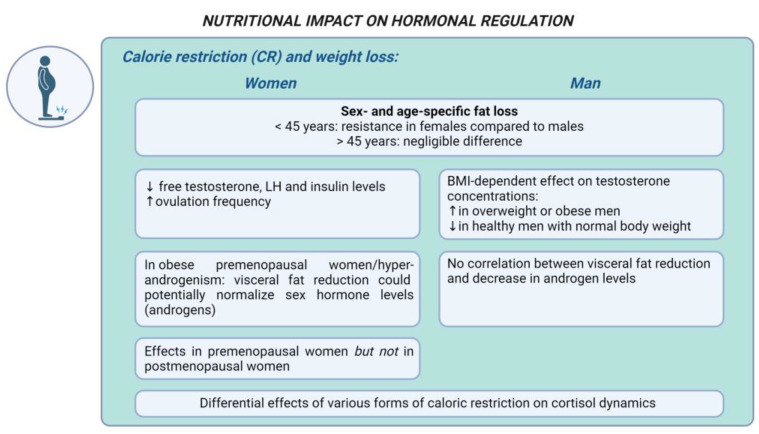
Representation of the main gender-specific nutritional impacts on hormonal balance in obesity-related endocrine disruptions, considering calorie restriction and weight loss. ↑ increase, ↓ decrease.

Figure 2 represents the main gender-specific nutritional impacts on hormonal balance in obesity-related endocrine disruptions, considering CR and weight loss.

*Western diet and high-fat diets.* The Western diet (WD) has been extensively discussed in nutritional science. This contemporary dietary pattern is characterized by a high consumption of processed and refined foods, red and processed meats, added sugars, and saturated and trans fats, along with a low intake of fruits, vegetables, whole grains, and nuts [127], in addition to a high intake of ultra processed foods (UPFs) and high glycemic index (GI) foods [128]. This diet has been linked to several chronic diseases, including obesity, T2D, CVDs, and certain cancers [128]. The WD significantly affects cortisol levels in obese individuals, both males and females. The diet’s high consumption of processed foods, saturated fats, and sugars leads to chronic inflammation and oxidative stress, disrupting the HPA axis [6]. This disruption results in elevated cortisol levels, especially in response to stress, worsening metabolic issues and facilitating additional weight gain. In obese individuals, the WD exacerbates cortisol secretion due to factors like increased adipose tissue, IR, and hormonal signaling disruptions, ultimately advancing complications related to obesity [129]. Furthermore, diets high in processed meats, soy, potatoes, full-fat dairy, coffee, alcohol, sugary drinks, and sweets have been linked to adverse reproductive health outcomes [130]. The WD is typically deficient in antioxidant-rich foods and abundant in pro-oxidant substances, such as SFAs and advanced glycation end products (AGEs). For this reason, the WD may contribute to hormonal imbalances in both sexes through OS [131]. Studies have shown that individuals adhering to the WD exhibit lower levels of antioxidant molecules such as vitamin C, vitamin E, and beta-carotene compared to those following a healthy diet [132,133]. Additionally, the recent literature highlights that the WD may contribute to increased systemic inflammation due to its high fat content, as well as affecting OS activation in both sexes [134]. In Hill’s study [135], following a WD resulted in elevated plasma cholesterol levels in men. In addition, this dietary shift was associated with significant reductions in plasma testosterone levels [135]. A recent cross-sectional study conducted on 125 Taiwanese men reported that greater adherence to a typical high-calorie WD was associated with a decrease in testosterone levels by 0.87 ng/mL [136]. Furthermore, a recent meta-analysis by Whittaker and Harris [137] revealed that very-high-protein diets (>3.4 g/kg/day) are associated with a decrease in men’s total testosterone levels by approximately 5.23 nmol/L. This suggests that adopting hyper-protein diets for weight loss or muscle mass gain might be counterproductive, potentially adversely affecting testosterone levels. Kulkarni et al. [138] observe an 89% prevalence of hirsutism in urban women consuming a WD, compared to 22% in rural women following a vegetarian diet. The WD may play a causal role in the development of hyperinsulinemia and IR prior to weight gain, as they lead to elevated levels of both insulin and OS [139]. The findings from a study suggest that childhood exposure to a WD might induce hormonal imbalances during the prepubertal reproductive maturation phase, potentially leading to persistent ovarian dysfunction throughout life [138]. Lin and Lujan found that the increased carbohydrate intake in women consuming a WD affects FSH, a pivotal hormone for follicular growth [140]. Similarly, protein intake, total energy intake, and cholesterol levels influence the production of androstenedione, a precursor to testosterone [140]. It has been shown that a WD can reprogram the innate immune system and induce low-grade inflammation [141]. The WD lead to increased postprandial insulin production and may result in insulin oversecretion and hyperinsulinemia, promoting fat accumulation, inhibiting lipolysis, and causing increased appetite, hyperphagia, and weight gain [121,139]. An increasing body of evidence suggests that GM is adversely affected by the WD, promoting inflammation due to both structural and behavioral changes in the resident bacteria, and these alterations are associated with obesity and subsequent hormonal changes [142].

A high-fat diet (HFD) is characterized by a lipid intake constituting more than 30% of the total energy consumption, with some studies reporting intakes as elevated as 45% to 60% [143]. HFDs, regardless of their role in obesity development, have been shown to impair the function and reproductive capacity of the hypothalamic–pituitary–ovarian (HPO) axis in women [144]. The prolonged consumption of a HFD in women can lead to the accumulation of abdominal adipose tissue, induce IR, and stimulate testosterone production from circulating androgens, thereby suppressing gonadotropin secretion [145]. The detrimental impact of HFDs primarily stems from their effect on the insulin signaling pathway [145]. Similarly, a review noted that in studies involving overweight and women with obesity with PCOS, HFDs were associated with increased IR and a reduced follicular response to FSH, leading to impaired follicular development [146]. Most observational studies have identified associations between dietary fatty acid intake and androgen concentrations in men. HFDs are inversely associated with total testosterone levels, although the impact varies depending on the type of fatty acids consumed [147]. A study showed that a higher intake of polyunsaturated fatty acids (PUFAs) was associated with lower testosterone concentrations, while an increased intake of SFAs and monounsaturated fatty acids (MUFAs) correlated with elevated testosterone levels in males [148]. However, findings are not consistent across all studies. In another study involving 209 men, a higher intake of MUFAs and TFAs was associated with lower testosterone levels [147]. A HFD appears to involve reducing Bifidobacterium spp. and overactivating the endocannabinoid system. These events can unfavorably alter the normal intestinal microbial composition, leading to increased intestinal permeability, as evidenced by reduced and disorganized tight junction proteins like zonulin and occludin in the colon [149]. Similarly, a HFD promotes the overgrowth of Gram-negative pathogens, facilitating LPS absorption through the intestinal barrier [150]. The LPS translocation primarily caused by a HFD is associated with the low-grade systemic inflammation induced by obesity [151]. In conclusion, both the WD and HFD have significant implications for hormonal balance, systemic inflammation, and metabolic health. These dietary patterns, characterized by the high consumption of processed foods and SFAs and the low intake of antioxidants, have been associated with hormonal imbalances, including reduced testosterone levels, IR, and impaired reproductive function.

**Figure 3:**
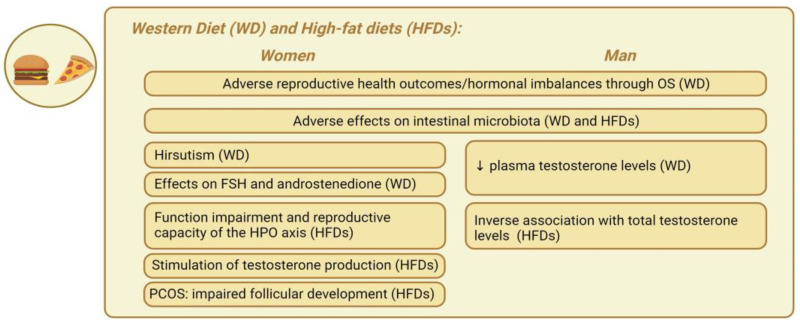
Representation of the main gender-specific nutritional impacts on hormonal balance in obesity-related endocrine disruptions, considering the Western diet and high-fat diets. ↓ decrease.

Figure 3 represents the main gender-specific nutritional impacts on hormonal balance in obesity-related endocrine disruptions, considering the WD and HFDs.

*Low-carbohydrate diets and ketogenic diets*. Low-carbohydrate (low-CHO) diets restrict carbohydrate (CHO) intake, with carbohydrates comprising less than 10% of total energy in very-low-carb diets (VLCDs) or 20–50 g/day in ketogenic diets (KDs). Low-carb diets include those with less than 26% or fewer than 130 g/day of CHOs, while moderate-carb diets range from 26% to 44% [152]. The KD is characterized by a high-fat, adequate-protein, and low-CHO composition [153]. Due to limited CHO availability, the body shifts from metabolizing CHOs to fats for energy production [153]. The liver converts fats into fatty acids and produces ketone bodies, which replace glucose as the primary energy source. This accumulation of ketones in the blood is also known as nutritional ketosis. Induced nutritional ketosis through a KD leads to the synthesis of ketone bodies, such as acetoacetate, acetone, and beta-hydroxybutyrate, which are quantifiable in serum or urine [153]. Nutritional ketosis generally raises serum ketone levels from 1 mmol/L to 7 mmol/L without causing metabolic acidosis [154]. In KD studies, postprandial responses in women exhibited higher levels of beta-hydroxybutyrate compared to men, potentially due to differences in the adipose tissue distribution and estrogen-mediated lipid metabolism, highlighting a gender-specific metabolic response that needs further investigation [155]. In obese patients of both genders, the KD treatment has demonstrated greater weight loss compared to other balanced diets, positioning it as an alternative tool against obesity [153,156]. This increased weight loss may result from enhanced satiety due to protein’s greater filling effect, direct appetite suppression by the KD, and shifts in the circulating levels of appetite-regulating hormones such as ghrelin and leptin [157]. Other suggested mechanisms include decreased lipogenesis, heightened lipolysis, reduced resting respiratory quotient, the increased metabolic costs of gluconeogenesis, and the thermic effect of proteins [153,156]. Recent evidence suggests that reducing CHO intake can decrease circulating insulin levels and improve hormonal imbalance in obese individuals of both genders [158]. A recent meta-analysis found that short-term (<3 weeks) low-CHO diets moderately increased resting cortisol levels, but long-term (≥3 weeks) low-CHO diets had no consistent effect on resting cortisol in males [159].

Numerous studies have indeed shown that low-CHO diets not only induce rapid and significant weight loss but also reduce serum insulin, thereby improving insulin sensitivity [160]. The subsequent biochemical cascade fosters a more favorable hormonal equilibrium, characterized by a reduction in free testosterone and an elevation in sex hormone-binding globulin in women [160]. This shift may correlate with enhanced menstrual function and fertility. The combined advantages of weight loss and hormonal balance further optimize clinical outcomes in women both with and without PCOS, ultimately enhancing fertility [161]. Nonetheless, it is crucial to acknowledge that CR may hold greater significance than macronutrient composition [162]. The metabolic and endocrine effects of a KD in women with obesity are characterized by improvements in body weight, free testosterone levels, the luteinizing hormone/follicle-stimulating hormone ratio, and fasting insulin levels [153,163]. A KD leads to decreased androgen secretion and increased sex hormone-binding globulin, enhancing insulin sensitivity and thereby normalizing endocrine functions. This dietary intervention and lifestyle management has beneficial effects in treating PCOS patients with obesity and T2D. The CHO intake also influences male sex hormones. Low-CHO diets decrease plasma total testosterone levels, while high-CHO diets can increase them [117,158]. However, the effect of CHOs on androgen levels may differ between genders, and the increased consumption of refined CHOs is associated with decreased SHBG levels in both men and women [158]. A recent study aimed to explore the metabolic dynamics and factors influenced by these low-CHO diets, particularly focusing on sex differences and their effects on specific metabolic hormones and adipose tissue compartments in mice [164]. A KD led to a temporary increase in TNFɑ only in male mice and had no impact on behavior. Intriguingly, these findings suggest that women may be more susceptible to the unfavorable metabolic effects of low-CHO diets [164]. Significant sexual dimorphism was observed, with reduced body weight and subcutaneous fat only in male mice on a KD. Therefore, a KD resembling fasting induces a notable decline in serum T3 hormone levels [165]. This reduction frequently accompanies an elevation in reverse T3 hormone, which correlates strongly with ketone body presence [165]. Nevertheless, there is inconclusive and conflicting data concerning the potential adverse effects of a KD on thyroid function. In 2022, Iacovides et al. conducted a randomized crossover study with two isocaloric diets: a low-CHO diet and a Very-Low-Calorie Ketogenic diet (VLCKD) [166]. Following both diets, the plasma levels of TSH and T4 remained stable. However, T3 plasma concentrations decreased more markedly with the VLCKD than the low-CHO diet (*p* = 0.003) and were associated with a more significant body mass reduction [166]. The study indicates that nutritional ketosis may alter the circulating T3:T4 ratio, leading to an elevation in inactive T4 and a decline in active T3 without altering the TSH levels. The mechanisms underlying these hormonal shifts remain to be clarified [166]. The VLCKD can act as a stressor, triggering increased cortisol production through the activation of the HPA axis [167]. Additionally, it remains unclear to what extent CHO restriction should be applied or for how long it should be maintained to achieve optimal results. A long-term KD results in glucose intolerance associated with inadequate insulin secretion, insulin resistance, and reduced beta and alpha cell mass in mice [168].

**Figure 4:**
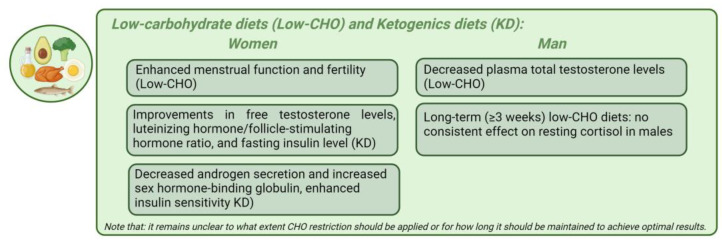
Representation of the main gender-specific nutritional impacts on hormonal balance in obesity-related endocrine disruptions, considering low-carbohydrate diets and ketogenic diets.

Figure 4 represents the main gender-specific nutritional impacts on hormonal balance in obesity-related endocrine disruptions, considering low-CHO diets and KDs.

Plant-based diet. A plant-based diet (PBD) refers to any dietary pattern that emphasizes the consumption of plant-derived foods while excluding most or all animal products [169]. PBDs include both vegan and lacto–ovo–vegetarian diets and are increasingly popular in the Western world due to health and environmental concerns. A healthy PBD consists of unprocessed plant foods such as fruits, vegetables, whole grains, legumes, nuts, and seeds, while an unhealthy PBD includes refined grains and added sugars [169]. PBDs have been shown to be rich in micronutrients but less energy-dense [170]. The only nutrient commonly deficient when eliminating animal products is vitamin B12, which may require supplementation [169,170]. Calcium is another nutrient to consider; however, supplementation is not recommended due to the potential increased risk of CVD [171]. PBDs have been linked to improved gut microbiota symbiosis and increased insulin sensitivity in both genders [172]. In the Adventist Health Study-2, a PBD was found to be associated with a reduced risk of hypothyroidism compared to an omnivorous diet, particularly in women [173]. Among premenopausal women adhering to the recommended dietary intake for total protein, a reduced consumption of plant-based proteins, as opposed to animal proteins, was linked to compromised ovulatory function, underscoring the significance of a sufficient plant protein intake for reproductive health [174]. In addition to its influence on cardiometabolic health, the dietary protein intake has also been linked to reproductive disorders. An elevated intake of animal proteins correlated with a higher risk of ovulatory infertility, while a protective role was suggested for the plant protein intake [175]. One source of protein in PBDs is soy, which has raised concerns among some researchers due to its potential negative effects on the endocrine system, particularly through isoflavones, as highlighted in previous animal studies [176]. A meta-analysis involving 648,913 participants from 16 prospective cohort studies found that the high consumption of soy foods was associated with a 13% reduction in breast cancer risk [177]. Another recent meta-analysis [178] investigated the impact of soy-derived phytoestrogens (isoflavones) on male hormonal health, addressing concerns that they might adversely affect male hormone levels by mimicking estrogenic activity. This study found no significant influence of soy protein or isoflavone intake on measured hormonal parameters. The subgroup analyses based on isoflavone dosage and study duration similarly showed no significant effects. Consequently, the meta-analysis concluded that neither soy nor its isoflavones alter total testosterone, free testosterone, estradiol, estrone, and SHBG levels in men, irrespective of dosage or study duration [178]. A noteworthy focus lies in the distinct role of plant proteins in weight management and metabolic health. In obese individuals, plant proteins, specifically the higher intake of non-essential amino acids, are associated with down-regulated insulin secretion and increased glucagon secretion [179]. This leads to the stimulation of gluconeogenesis, hepatic lipid oxidation, and lipolysis and a decrease in both IGF-1 synthesis and cholesterol levels [179]. Hepatic lipid oxidation promotes appetite control and lowers the respiratory quotient, potentially playing a role in weight reduction, further supported by the thermogenic effect of glucagon. Human adipocytes express IGF-1 receptors, so the downregulation of IGF-1 activity can also promote leanness [179]. The essential amino acids in plant proteins promote a higher net glucagon activity compared to an omnivorous diet, favoring weight loss and reducing LDL cholesterol in obese individuals [179]. In conclusion, PBDs offer several health benefits, including improved cardiometabolic health, weight management, and reduced disease risks, particularly in obese individuals and women. Despite concerns about specific nutrients, recent research provides reassuring evidence on the safety of soy. However, further studies are needed to better understand the role of plant proteins in metabolic health and their differential effects between genders. Overall, PBDs remain a promising dietary choice for enhancing overall health.

**Figure 5:**
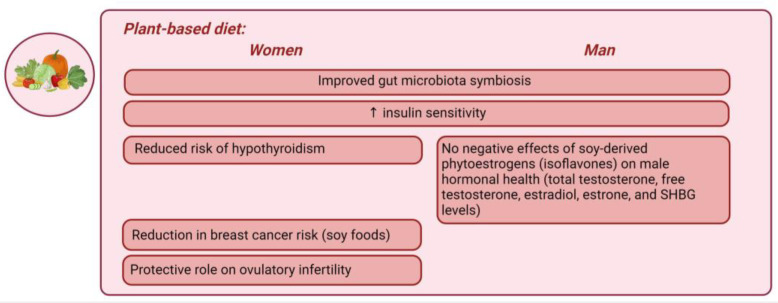
Representation of the main gender-specific nutritional impacts on hormonal balance in obesity-related endocrine disruptions, considering plant-based diets. ↑ increase.

Figure 5 represents the main gender-specific nutritional impacts on hormonal balance in obesity-related endocrine disruptions, considering PBDs.

*Mediterranean diet.* The traditional Mediterranean diet (Med-Diet) represents a culinary heritage deeply rooted in Mediterranean cultures, emphasizing the consumption of olive oil, fruits, vegetables, and grains sourced from local small- and medium-sized agricultural enterprises, complemented by the moderate intake of fish and dairy and the limited consumption of red meat and red wine. This dietary pattern, reflecting cultural practices and traditional dietary norms, is characterized by its low saturated fatty acid content and high concentration of antioxidants and fiber [180]. Notably, it features significant quantities of monounsaturated fatty acids, primarily from extra virgin olive oil (EVOO), and omega-3 PUFAs derived from fish and nuts. Extensive evidence supports the inclusion of the Med-Diet among dietary strategies highly beneficial for preventing major non-communicable diseases [180,181,182]. The Med-Diet provides high levels of phytochemicals, such as dietary polyphenols and flavonoids, known to have beneficial biological effects, including antioxidative, anti-inflammatory, immunomodulatory, antitumoral, antidiabetic, and anti-obesity activities [183]. This dietary pattern, intricately woven into cultural practices and traditional dietary norms, holds the key to hormonal balance modulation, particularly in the context of obesity and metabolic disruptions. Data from the INTERCATH study indicated a significant correlation between adherence to the Med-Diet and levels of high sensitivity C-reactive protein (hs-CRP), a marker of inflammation associated with an increased risk of CVDs and other chronic conditions [184]. The Med-Diet is associated with improved reproductive health outcomes, particularly in mitigating the negative effects of an inadequate diet, obesity, and metabolic imbalances [26,34,46,180]. While direct evidence on its impact on male fertility is limited, studies suggest that the Med-Diet may enhance fertility by improving the quality, count, and conception rates of seminal fluid [185]. A diet rich in vegetables, fruits, and whole grains has been associated with increased seminal fluid volume and motility, aided by bioactive compounds that support mitochondrial function and reduce oxidative stress [186]. For example, resveratrol, found in grapes, exhibits antioxidant and anti-inflammatory properties [186,187]. PUFA n-3 supports metabolic function, while olive oil supplementation can mitigate oxidative stress damage [186,187]. Additionally, the vitamins and other compounds in these foods further reduce oxidative stress. Long-term adherence to the Med-Diet, particularly the high polyphenol variant, is associated with a significant reduction in cortisol levels, suggesting potential cardiometabolic health benefits independent of weight loss [188]. The influence of the Med-Diet on the outcomes of assisted reproductive technologies (ARTs) is still under investigation, and, while evidence linking dietary choices to the risk of testicular cancer is inconclusive, certain associations with dietary fat, cheese, and fiber intake have been reported [189]. Recent studies have highlighted that diets rich in fish, shellfish, seafood, poultry, whole grains, vegetables, fruits, and low-fat dairy are positively associated with hormonal health outcomes in males [112,130]. Furthermore, the Med-Diet positively influences testosterone levels in men through its high antioxidant content and levels of polyunsaturated fats [190]. This dietary pattern modulates the glucose metabolism, potentially reducing insulin resistance and subsequently enhancing testosterone production. Additionally, the diet’s antioxidant-rich profile protects against ROS-induced oxidative damage, preserving seminal fluid quality and DNA integrity [191]. Furthermore, obese females adhering closely to the Med-Diet exhibit reduced inflammatory markers, highlighting its anti-inflammatory and metabolic properties. Studies have shown that adherence to the Med-Diet is associated with a reduction in the clinical severity of conditions such as PCOS, leading to decreased testosterone levels and improved body composition in affected women [187]. This underscores the potential therapeutic benefits of the Med-Diet in managing PCOS through its anti-inflammatory and metabolic properties. Furthermore, research indicates that obese females who adhere closely to the Med-Diet exhibit reduced inflammatory markers, including lower levels of hs-CRP, compared to their male counterparts. Gender differences in food choices within the Med-Diet may contribute to variations in its impact on inflammation [192]. Females consumed more plant-based foods and fewer fats and processed meats than males. The differing food choices within the Med-Diet between genders might explain the varied impact of this dietary pattern on inflammation [192]. Within a hypocaloric regimen, the Med-Diet has been shown to be more effective in improving IR in obese individuals compared to other low-energy dietary approaches, independent of calorie restriction [193]. Additionally, the reduction in insulin levels and other IR measures, such as the homeostatic model assessment (HOMA) index, induced by this dietary approach are both early and sustainable over time [193]. In conclusion, the Med-Diet emerges as a holistic dietary approach to optimizing hormonal balance and reproductive health, offering a beacon of hope in the fight against obesity-related endocrine disruptions. Its gender-specific impacts underscore the need for tailored dietary interventions to harness its full potential in promoting health and well-being.

**Figure 6:**
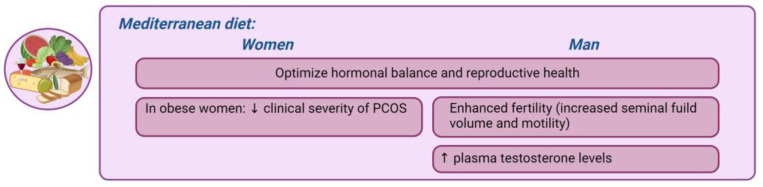
Representation of the main gender-specific nutritional impacts on hormonal balance in obesity-related endocrine disruptions, considering the Mediterranean diet. ↑ increase, ↓ decrease.

Figure 6 represents the main gender-specific nutritional impacts on hormonal balance in obesity-related endocrine disruptions, considering the Med-Diet.

## 7. Relevance of Future Research

This exploration of nutritional influences on hormonal balance and of the gender-specific implications for obesity-related endocrine disruptions represents a critical avenue for future research. Understanding the intricate interplay between diet, hormonal regulation, and gender-specific factors is paramount for developing targeted dietary interventions and therapeutic strategies to mitigate obesity-related endocrine disruptions and improving metabolic health in both men and women.

Research on gender transition represents a crucial frontier in understanding the complex interplay between hormones, metabolism, and dietary patterns. Individuals undergoing gender transition often experience significant hormonal changes as part of their medical transition process, which underscores the importance of personalized dietary interventions. These changes can have profound effects on metabolic health and may necessitate adjustments in dietary habits to support overall well-being, emphasizing the need for tailored dietary recommendations. However, there is a notable gap in research addressing the specific dietary needs and challenges faced by individuals undergoing gender transition. Future studies in this area could provide valuable insights into optimizing dietary interventions to support hormonal balance and metabolic health during the transition process. Moreover, such research could contribute to greater inclusivity in healthcare practices, ensuring that dietary recommendations are tailored to the diverse needs of transgender and gender-nonconforming individuals. By addressing these gaps in knowledge, researchers can facilitate the development of evidence-based dietary guidelines that promote optimal health outcomes for individuals across the gender spectrum.

Future investigations should delve deeper into elucidating the specific mechanisms by which different dietary patterns modulate hormonal balance, taking into account gender-specific differences. While calorie restriction has shown promising results in improving hormonal balance and metabolic health, particularly in overweight and obese individuals, its efficacy may vary based on age, gender, and baseline metabolic status. Additionally, the potential negative impacts of calorie restriction, such as nutrient deficiencies and metabolic adaptation, should be carefully considered, especially in the context of gender-specific metabolic differences.

Furthermore, the WD and HFDs have been associated with hormonal imbalances, systemic inflammation, and metabolic dysfunction, with gender-specific variations in response to these dietary patterns. While these diets contribute to obesity and endocrine disruptions, they also offer convenience and palatability, which may pose challenges for individuals attempting to adopt healthier dietary patterns, highlighting the importance of personalized dietary guidance tailored to individual needs and preferences, including gender-specific considerations.

Low-CHO diets and a KD have emerged as alternative approaches for obesity management, demonstrating rapid weight loss and improvements in insulin sensitivity, but their suitability and long-term effects may vary based on gender-specific metabolic differences. However, concerns have been raised regarding their long-term sustainability, potential adverse effects on thyroid function, and variability in individual responses, underscoring the need for personalized dietary recommendations.

A PBD offers several health benefits, including improved cardiometabolic health and reduced disease risks, particularly in obese individuals and women. However, they may be deficient in certain nutrients such as vitamin B12 and require careful planning to ensure adequate nutrient intake, with gender-specific considerations regarding nutrient needs and absorption efficiency.

The traditional Med-Diet stands out for its protective effects against CVDs and its potential to improve hormonal balance and reproductive health in both men and women. Rich in monounsaturated fats from olive oil, antioxidants from fruits and vegetables, and omega-3 fatty acids from fish and nuts, the Med-Diet offers a holistic approach to optimizing hormonal balance and metabolic health. Moreover, research has indicated that adherence to the Med-Diet is associated with reduced inflammation and improved reproductive health outcomes, particularly in women. However, while it emphasizes healthy fats and antioxidant-rich foods, its efficacy may be influenced by individual adherence and cultural preferences, highlighting the importance of considering gender-specific dietary habits and preferences in dietary recommendations.

## 8. Conclusions

In conclusion, while various dietary patterns offer potential benefits for hormonal balance and metabolic health, there is no one-size-fits-all approach. Future research should aim to identify personalized dietary interventions tailored to individual metabolic and hormonal profiles, considering both the benefits and limitations of different dietary patterns. This personalized approach could optimize the effectiveness of dietary interventions for obesity management and metabolic health improvement, particularly in the context of gender-specific differences. By integrating gender-specific considerations into dietary recommendations, healthcare professionals can better support individuals in achieving optimal health outcomes across the gender spectrum.

## Figures and Tables

**Table 1:** Summary of the body composition and fat distribution differences in man and women and the main endocrine and hormonal imbalances that occur as a result of obesity in men and women.

	In Females	In Males
Body composition and fat distribution	Body fat percentage of 15%, with the total adipose tissue volume being 12% of the total body weight [17] Gluteofemoral fat and then, after menopause, abdominal fat [27] Essential fat of 5–9%, encompassing sex-specific fat [22]	Body fat percentage of 12%, with the total adipose tissue volume being 4% of the total body weight [17] Abdominal fat [22] Greater visceral adiposity → increased postprandial insulin, free fatty acids, and triglycerides [28]
**Hormonal Imbalances**	**In Females**	**In Males**
Obesity	Obesity → irregular menstrual cycles, miscarriage rates, anovulation, and adverse neonatal and maternal outcomes [68]; hirsutism [69]; compromission of oocyte quality and development [78]; prolonged gestational periods and gestational diabetes [79]; and higher androgen levels [64] Higher total and free testosterone levels but lower levels of androstenedione and SHBG [64] → visceral white adipose tissue hypertrophy and/or hyperplasia → risk of IR and metabolic syndrome in hyperandrogenic women Adipose tissue and imbalance of adipokine levels → PCOS	Adipocyte hypertrophy and hyperplasia through adipokine secretion [34] → impact on HPG axis [36] Relationship between thyroid hormones, leptin, and reproductive function → male infertility [41] Erectile dysfunction and elevated scrotal temperature → male infertility [42] Testosterone conversion to estradiol → secondary hypogonadism [50]
Hyperinsulinemia, leptin, and insulin resistance (IR)	Reproductive age: greater insulin sensitivity, increased stimulated insulin secretion, and lower fasting glucose and HbA1c levels [86] During menopause: increased blood pressure and LDL cholesterol and HbA1c levels → body fat distribution → impaired glucose tolerance (IGT) [86] Increased insulin stimulates theca cells to produce androgens → extradiol synthesis [80] PCOS: increased insulin → androgen production, contributing to follicular arrest and anovulation and inhibition of SHBG production → increased testosterone [89] PCOS: increased insulin → enhancing LH pulse amplitude, increasing sensitivity to gonadotropin-releasing hormone [90] → P450c17α enzyme → increasing adrenal androgen production → hyperandrogenism [91] Increased leptin → imbalance of plasma female sex hormone levels [87] and disruption of oocyte development [88] Obesity-related hyperinsulinemia and IR → menstrual irregularities, hyperandrogenism, and alterations in steroidogenesis (also in non-obese) [92]	Elevated insulin → Decreased SHBG [44] and impact on fertility indicators [51] Increased leptin secretion → inhibition testosterone production [35] Fat mass → promotion of testosterone conversion to estradiol [44] → secondary hypogonadism [50] Low testosterone levels → bone density, fat distribution, muscle mass, red blood cell production [45,46], IR, and obesity [44]
Cortisol	↑ Cortisol in obesity → visceral fat accumulation, IR, and an increased risk of metabolic syndrome [95] Premenopausal women with obesity with elevated WHR and tendency to visceral fat accumulation → increased urinary cortisol and normal dexamethasone inhibition of cortisol secretion [96] Responses to physical or mental stressors → increasing serum cortisol levels, with no influence on prolactin or GH [96] Androgynous obesity in women → increased sensitivity along HPA axis → increased cortisol secretion → contribution to abnormal fat depot distribution and metabolic abnormalities	↑ Cortisol → visceral fat accumulation, insulin resistance, and an elevated risk of metabolic syndrome [55] Stress input and elevated cortisol in obesity → hypothalamic–pituitary–adrenal (HPA) axis dysregulation and impact on reductions in IGF-I and testosterone levels [55]
Thyroid hormones	Fluctuation in TSH levels correlated to leptin concentrations [53,55,83] → thyroid dysfunction → IR, dyslipidemia, and cardiovascular complications [70] Altered thyroid function → impact on energy expenditure, fat metabolism, and weight management [71]	Increased TSH levels and triiodothyronine (T3), unchanged T4 levels [52,55] → thyroid hormone resistance → increased TSH and fT3 secretion Altered leptin production → impact on TSH regulation through leptin resistance [54] Positive correlation between BMI and TSH levels [55]
Dyslipidemia		Oxidative stress → quality of male germ cells and infertility Modification in structures and functions of testes and epididymis, energy metabolism, and spermatogenesis [18,46] Acrosomal response and capacitation → hormonal imbalances and erectile disfunction [18,46]
Oxidative stress and inflammation	Imbalance between the production of ROS and antioxidant defenses → compromission of follicle development, oocyte maturation, and embryo and placental growth [93] Disruptions in leptin during pregnancy → impediment of oocyte development [79] Menstrual irregularities, infertility, intrauterine growth restriction, preeclampsia, recurrent miscarriages, and premature births [95]	Elevated ROS levels in sperm Contribution to IR and leptin dysregulation [42,58]
Gut microbiota composition	PCOS → reduces alpha and beta diversity [96] Dysbiosis and Lactobacillus deficiency → interference with assisted reproduction treatments [98] Gram-negative bacteria, Chlamydia trachomatis, and Gardnerella vaginalis in cervical flora → infertility problems [97]	Increased lipopolysaccharides → promotion of obesity and IR [59], mediated by TNF-α, IL-6, and leptin from adipose tissue Dysbiosis → impact on blood–testis barrier development and intratesticular testosterone levels → Sertoli cell development and maturation [63]

↑ increase, → leads to.

## Data Availability

No new data were created or analyzed in this study. Data sharing is not applicable to this article.
